# Supplementary material for: Design of peptides with noncanonical amino acids using flow matching
Source: Bioinformatics. 2026 Aug 31;42(9):btag654. doi: 10.1093/bioinformatics/btag654 (PMC13577156; doi:10.1093/bioinformatics/btag654)
Supplement: Supplementary file 1 [file btag654_supplementary_data.pdf]

Supplementary Information

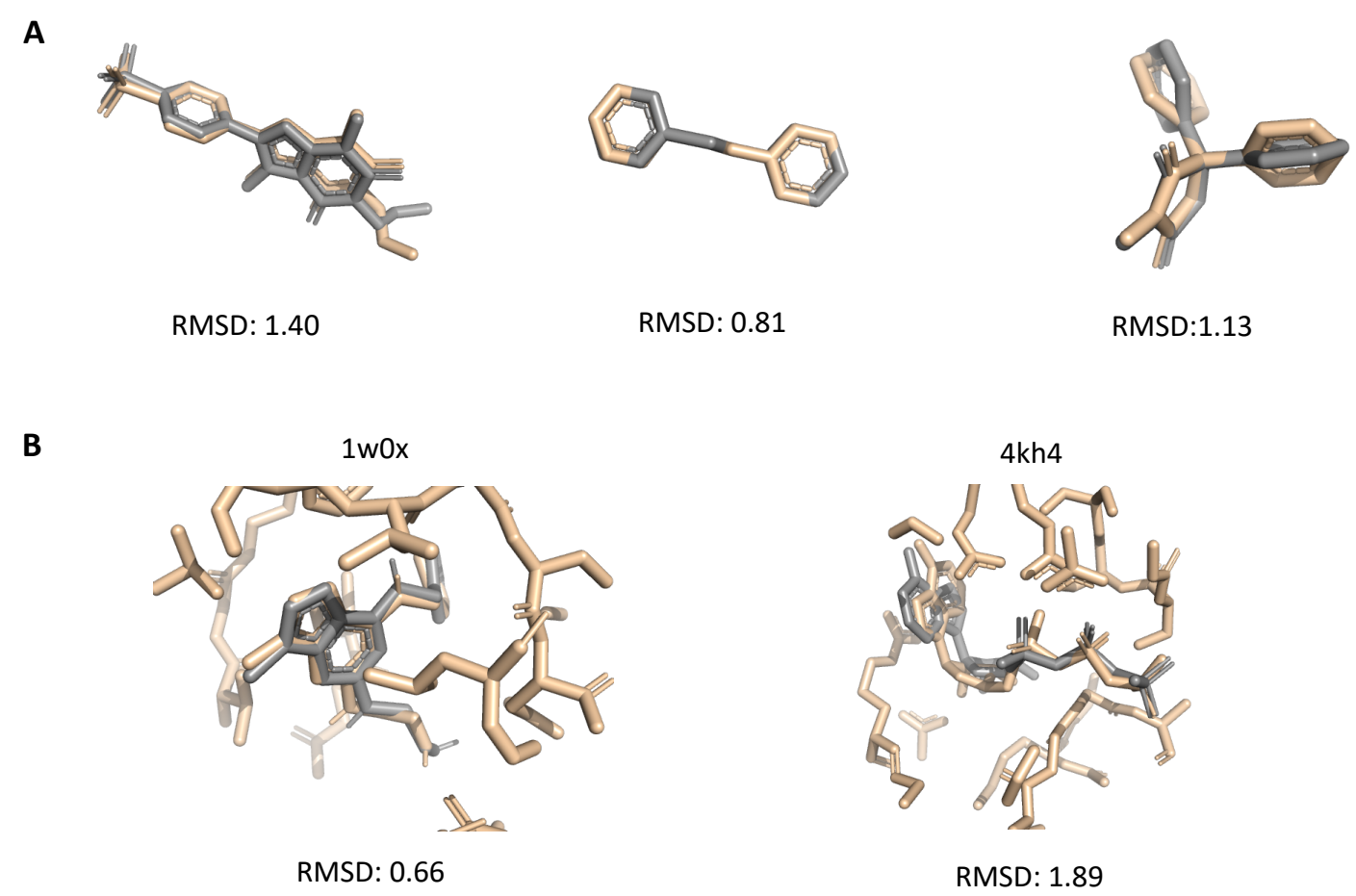

Figure S1: Randomly selected samples from pretrained models on (A) Pubchem and (B) Plinder datasets.

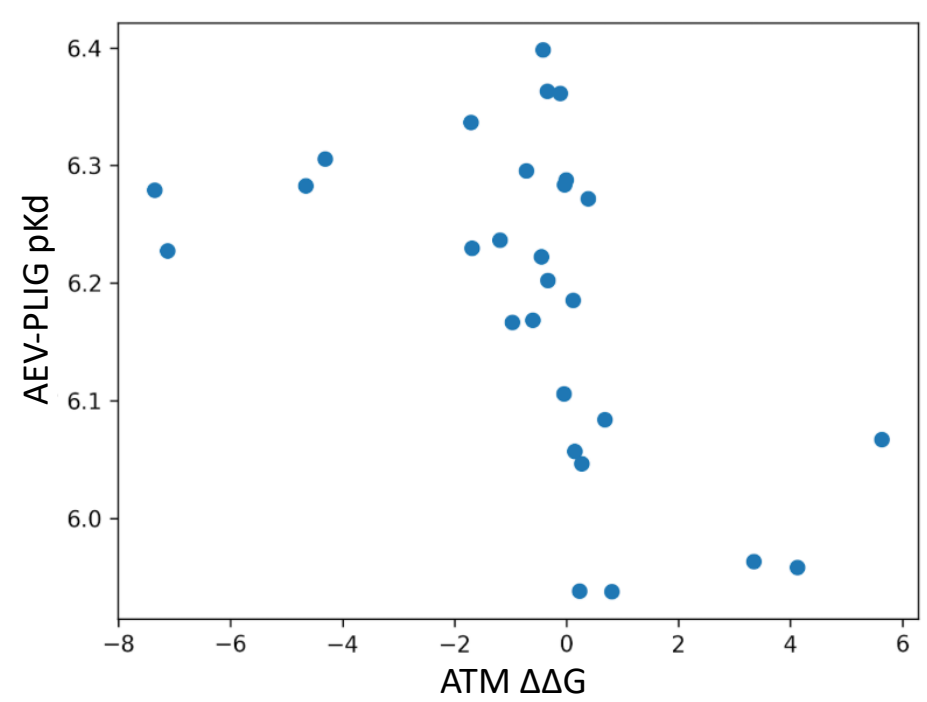

Figure S2: ATM $\Delta\Delta G$ and AEV-PLIG pK_d on PDB 1SSC with a smaller set of ncAAs from CycPeptMPDB (Li et al., 2023), exhibiting a Spearman correlation of -0.62 with p $<$ 0.01.

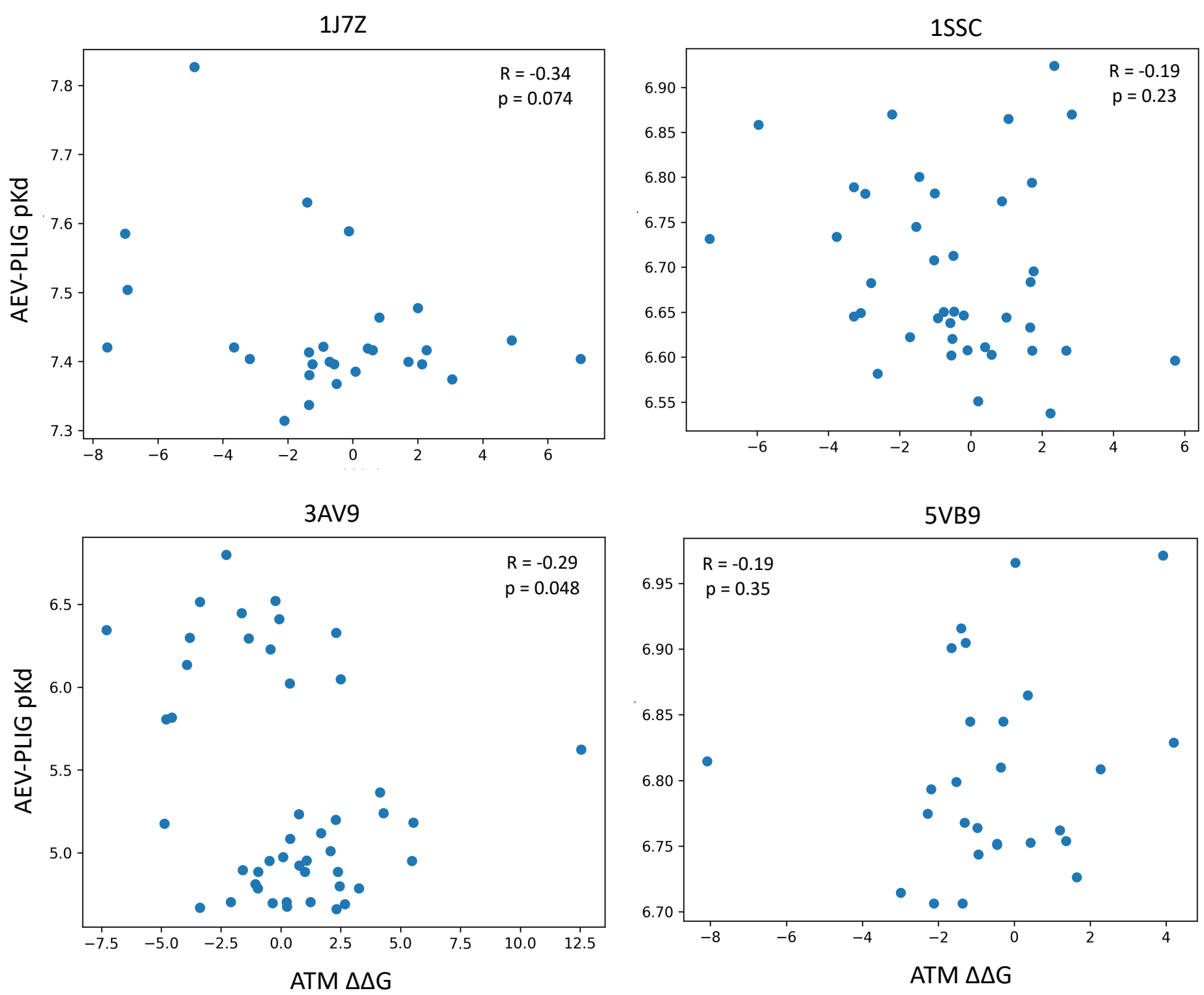

Figure S3: ATM $\Delta\Delta G$ and AEV-PLIG pK_d correlation on four test cases.

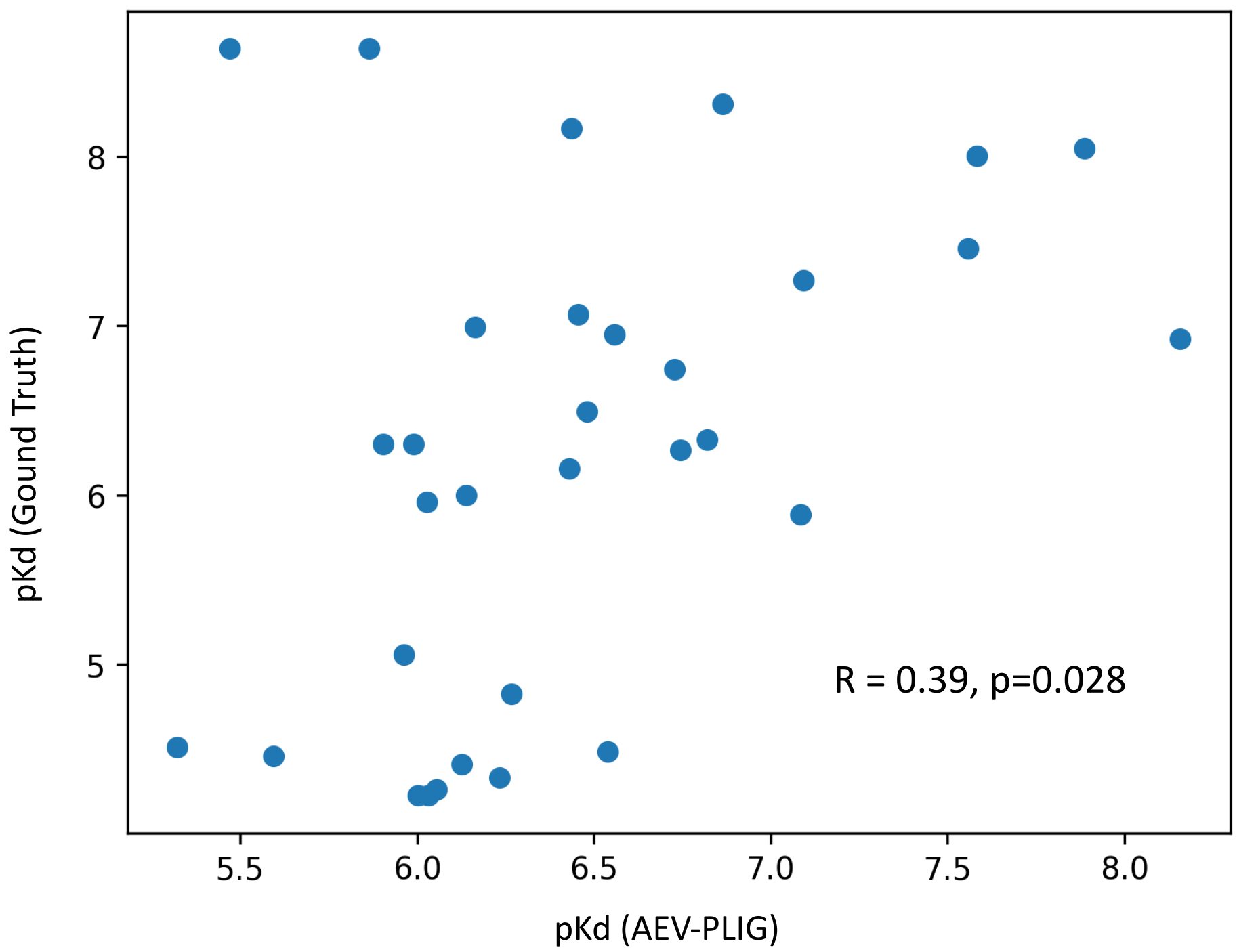

Figure S4: AEV-PLIG predictions vs experimental Kd values on long peptides (20-30aa) in PDBBind2020. These peptides are found in the PDBBind-PP dataset and are not in the training set of AEV-PLIG.

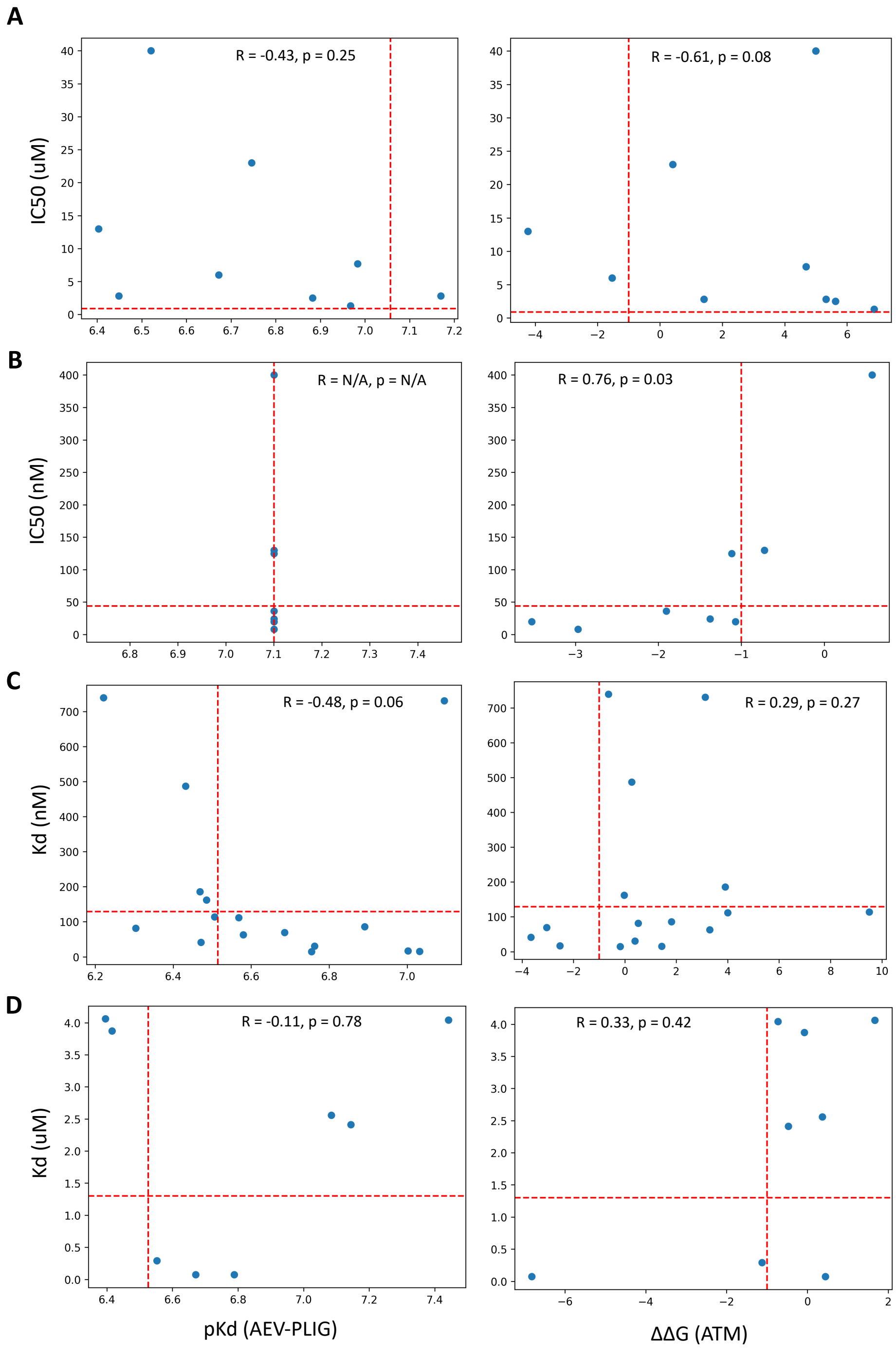

Figure S5: AEV-PLIG (left) and ATM (right) predictions of binding affinity plotted against experimentally measured binding affinities across the four experimental datasets in Table S5, corresponding to (A) Htra1-PDZ, (B) Mdm2-p53, (C) cetuximab-meditope, and (D) Undisclosed. Note that AEV-PLIG appears to predict the same affinity across all variants of Mdm2, perhaps due to

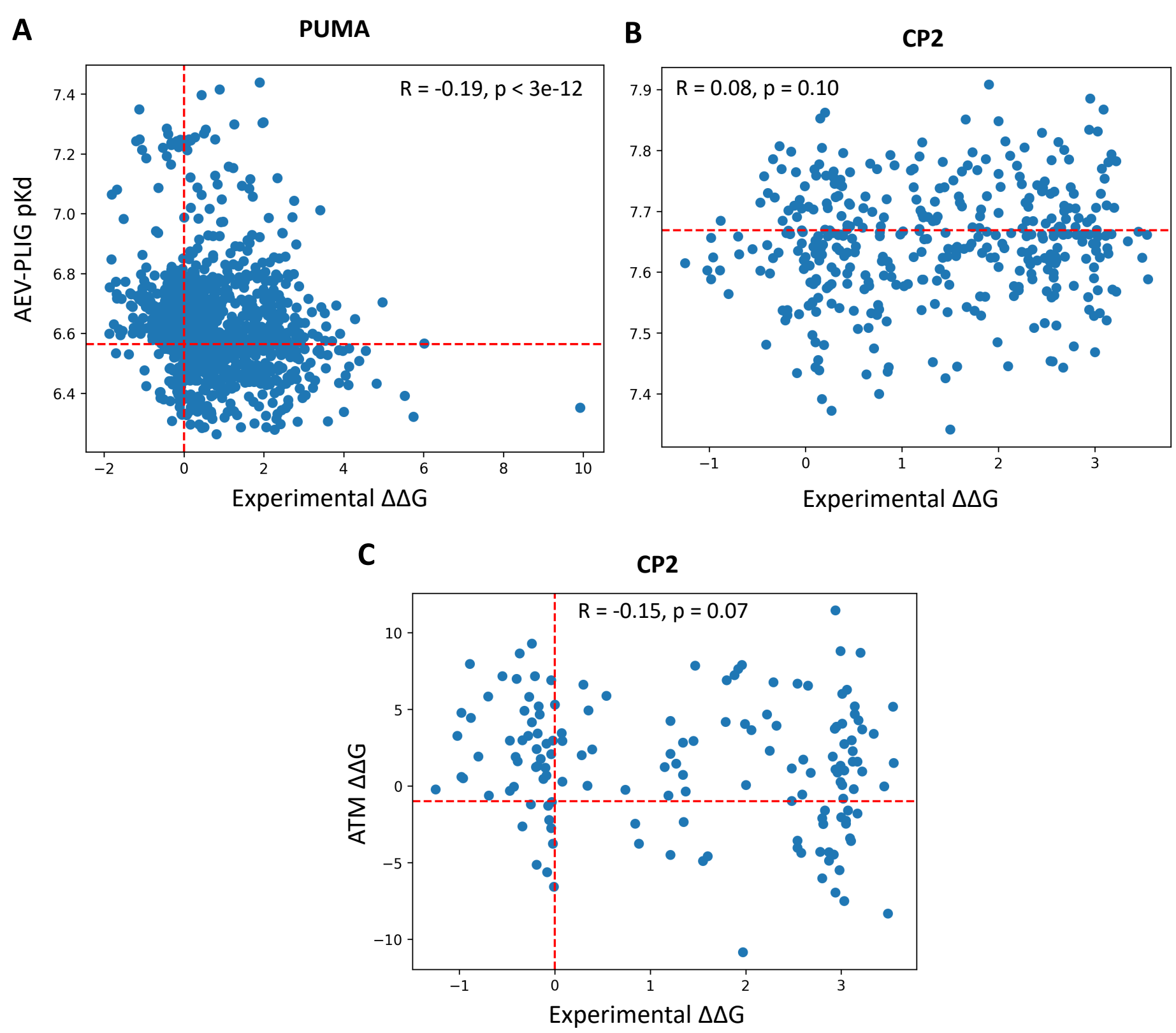

Figure S6: AEV-PLIG and ATM predictions of deep mutational scanning datasets (Rogers et al., 2018) on the peptides PUMA (A) and CP2 (B,C). ATM on the PUMA peptide was unsuccessful due to its length (35aa).

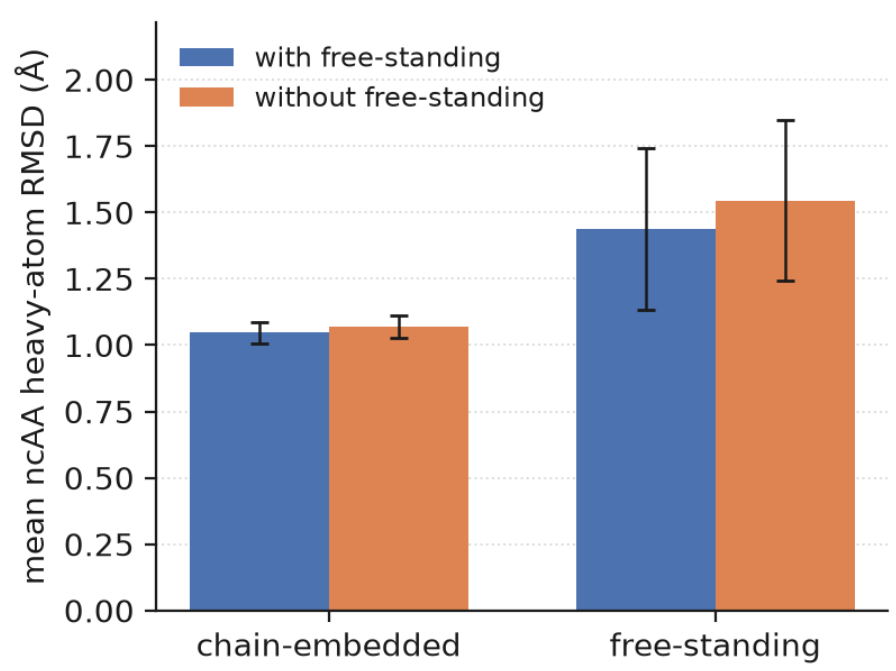

Figure S7: Free-standing vs. chain-embedded ncAA training data. Free-standing ncAAs (not peptide-bonded into a polypeptide chain) constitute 5.6% (2,177/38,618) of the stage-3 training examples and 3.0% (18/596) of the held-out test set. The plot shows the mean heavy-atom RMSD on the held-out test set for models finetuned with vs. without the free-standing examples: excluding them leaves performance on chain-embedded ncAAs (the setting of interest) essentially unchanged (1.05 vs. 1.07 Å) and only mildly degrades the small free-standing test set (1.44 vs. 1.54 Å, $n = 18$), indicating that these examples are harmless additional training data for chain-embedded prediction. Error bars denote the standard error of the mean.

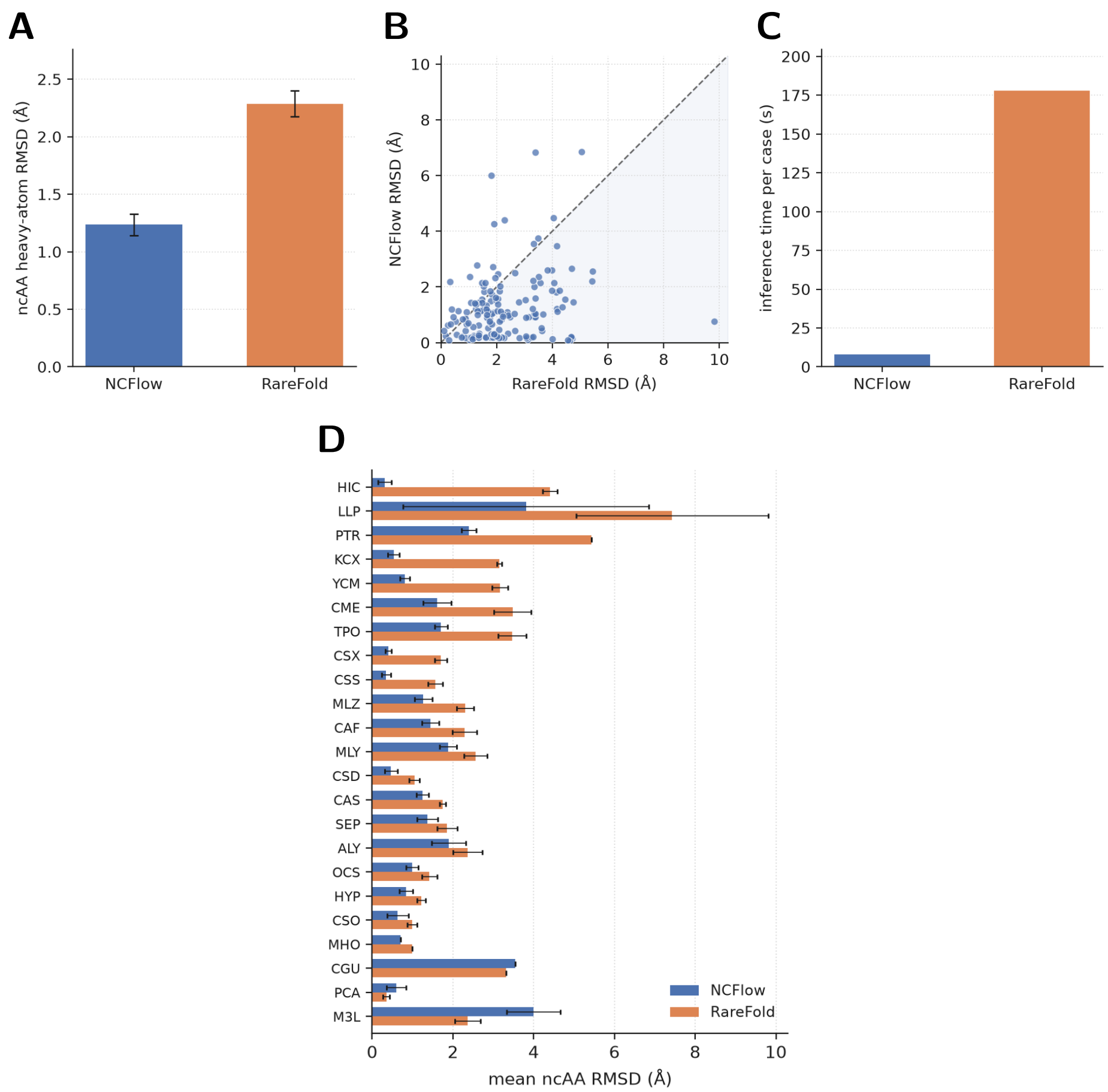

Figure S8: NCFlow vs. RareFold on a temporally held-out benchmark of 157 single-ncAA structures spanning 23 of RareFold's 29 supported ncAAs (PDB entries deposited after the 2023-07-28 training cutoff). (A) mean heavy-atom RMSD for each method's confidence-selected structure, using no ground-truth information: NCFlow, the highest-pLDDT of 8 flow samples (1.24 Å); RareFold, its single deterministic prediction (2.29 Å). NCFlow's best-of-8 oracle RMSD is 0.66 Å, and its 8 samples are still generated ∼22× faster than RareFold's single prediction (C). (B) per-case NCFlow (top-1-by-pLDDT) vs. RareFold RMSD; points below the dashed diagonal are cases where NCFlow is more accurate (123/157; Wilcoxon signed-rank $p < 10^{-14}$). (C) mean inference time per residue (7.9 s vs. 177.7 s; ∼22×). (D) per-code mean RMSD, with NCFlow lower in 22 of 23 codes. Error bars in (A) and (D) denote the standard error of the mean.

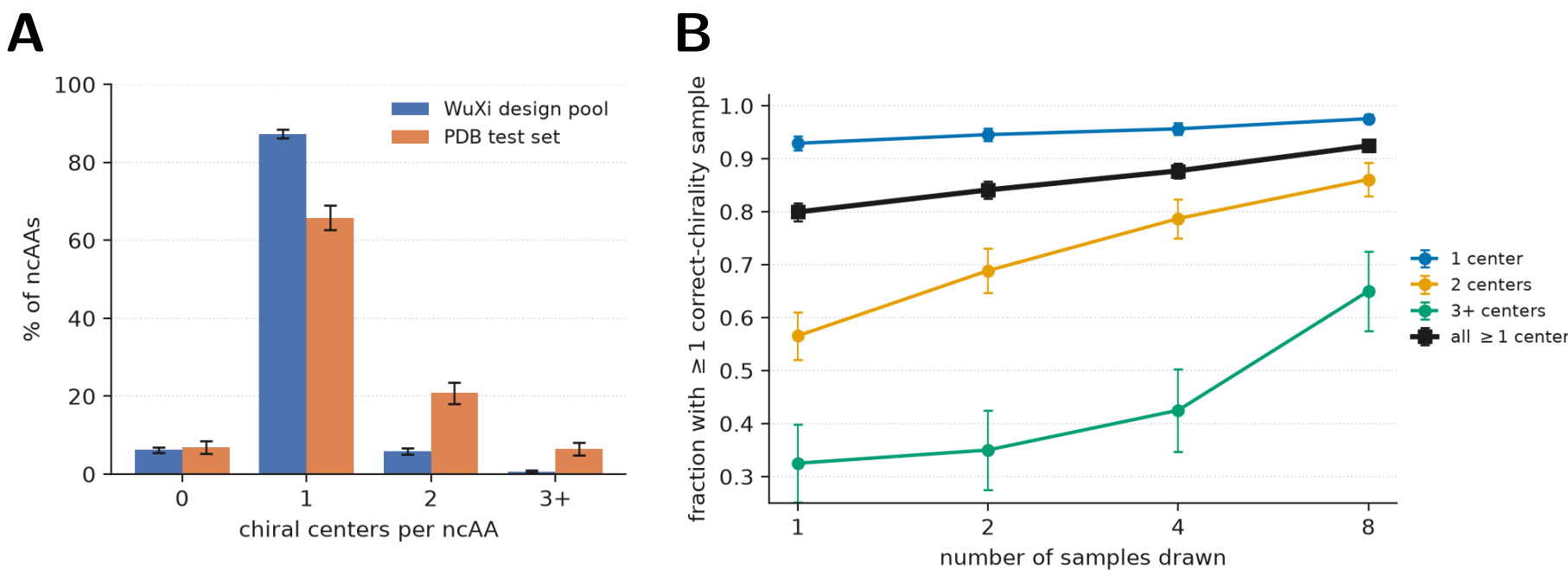

Figure S9: Chirality analysis. (A) number of chiral centers per ncAA for the 939-member WuXi AppTec design pool vs. the 231 unique PDB test ncAAs; the design pool is chirally simpler (mean 1.01 vs. 1.30 centers, with 87% of the pool having a single stereocenter). (B) fraction of test ncAAs with at least one chirally-correct sample as a function of the number of samples drawn, split by number of stereocenters. With 8 draws, 92.4% of ncAAs with at least one stereocenter are recovered (97.5%, 86.1%, and 65.0% for 1, 2, and $\geq$3 stereocenters). Error bars denote the standard error of the proportion.

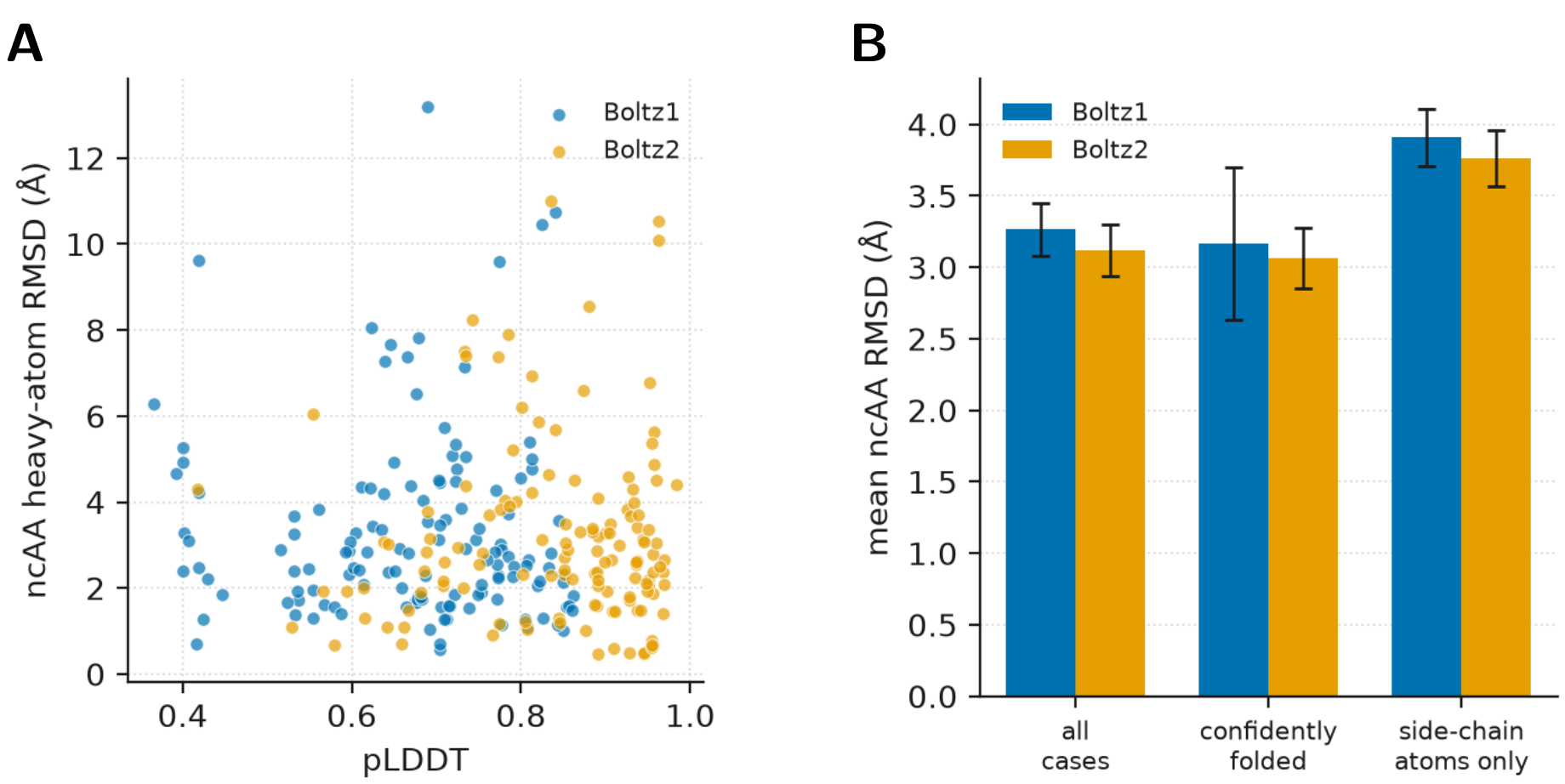

Figure S10: Controls for the task-difficulty mismatch in the Boltz comparison ($n = 136$ cases per model). (A) Boltz ncAA RMSD versus Boltz's own global fold confidence (complex pLDDT). The two are essentially uncorrelated (Spearman $\rho = -0.09$ for Boltz1 and -0.05 for Boltz2), so large ncAA deviations are not attributable to poorly folded scaffolds. (B) Mean ncAA RMSD over all cases, over the subset that Boltz itself folds confidently (complex pLDDT > 0.8; $n = 24$ and 96 for Boltz1 and Boltz2), and recomputed over side-chain atoms only (excluding N, CA, C and O). Restricting to confidently folded predictions barely changes the RMSD, and the side-chain-only comparison is worse, indicating that the discrepancy is concentrated in ncAA side-chain placement rather than in the surrounding scaffold. Error bars denote the standard error of the mean.

Table S1: Number of ncAAs per parent amino acid.

Parent AA	ncAA Count
LYS	111
PHE	379
PRO	35
ASN	9
MET	7
TRP	67
ARG	36
SER	23
TYR	63
CYS	49
GLY	57
GLU	17
THR	17
GLN	7
ALA	19
ASP	10
HIS	25
LEU	5
VAL	3
ILE	0
**Total**	979

Table S2: List of variants and corresponding binding affinities for HtrA1 PDZ domain (Runyon et al., 2007). Bold indicates the reference peptide used for relative binding affinity calculations.

ID	Sequence	IC50 (uM)
**H1C1**	**DSRIWWV**	**0.9**
H1C2	GWKTWIL	7.7
H1C3	DIETWLL	23
H1C4	WDKIWHV	2.8
H1C1c	DSRIWAV	6
H1C1d	DSRIAWV	40
H1C1e	DSRAWWV	13
H1C1f	DSAIWWV	2.5
H1C1g	DARIWWV	1.3
H1C1h	ASRIWWV	2.8

Table S3: List of variants and corresponding binding affinities for Mdm2-p53 (Phan et al., 2010). Bold indicates the reference peptide used for relative binding affinity calculations.

ID	Sequence	IC50 (nM)
**PDI**	**LTFEHYWAQLTS**	**44**
PDIQ	ETFEHWWSQLLS	8
6W11L	LTFEHWWAQLLS	20
1E6W	ETFEHWWAQLTS	20
6W8S	LTFEHWWSQLTS	24
6W	LTFEHWWAQLTS	36
6W9S	LTFEHWWASLTS	125
6W8S9S	LTFEHWWSSLTS	130
6N	LTFEHNWAQLTS	400
p53	ETFSDLWKLLPE	2000

Table S4: List of variants and corresponding binding affinities for cetuximab-meditope (van Rosmalen et al., 2017). Bold indicates the reference peptide used for relative binding affinity calculations.

ID	Sequence	Kd (nM)
**Md1**	**CQFDLSTRRLKC**	**129.4**
Md3	CQYNLSSRALKC	739
Md2	CVWQRWQKSYVC	731
Q1V	CVFDLSTRRLKC	111.5
S5G	CQFDLGTRRLKC	113.9
K10R	CQFDLSTRRLRC	81.3
Q1V_S5G	CVFDLGTRRLKC	69.4
Q1V_K10R	CVFDLSTRRLRC	63.2
S5G_K10R	CQFDLGTRRLRC	41.1
Q1V_S5G_K10R	CVFDLGTRRLRC	30.3
Q1V_D3N_S5G_K10R	CVFNLGTRRLRC	14.5
Q1V_S5G_T6I_K10R	CVFDLGIRRLRC	16.8
Q1V_D3N_S5G_T6I_K10R	CVFNLGIRRLRC	15.8
Q1V_S5Y_K10R	CVFDLYTRRLRC	86.3
Q1V_S5G_T6M_K10R	CVFDLGMRRLRC	162
Q1V_S5Y_T6M_K10R	CVFDLYMRRLRC	487
Q1V_D3R_S5G_K10R	CVFRLGTRRLRC	186

Table S5: Performance evaluation of AEV-PLIG and ATM binding affinity predictions on four protein-peptide variant datasets.

Target	Method	Accuracy	Precision	Recall
HtrA1 (Runyon et al., 2007)	AEV-PLIG	0.89	N/A	N/A
	ATM	0.78	N/A	N/A
	Both	**1.00**	N/A	N/A
Mdm2 (Phan et al., 2010)	AEV-PLIG	N/A	N/A	N/A
	ATM	**0.67**	**1.00**	**0.67**
	Both	**0.67**	**1.00**	**0.67**
Cetuximab (van Rosmalen et al., 2017)	AEV-PLIG	0.75	0.89	0.73
	ATM	**0.80**	0.50	**1.00**
	Both	0.44	**1.00**	0.18
Undisclosed[1]	AEV-PLIG	0.63	0.50	**1.00**
	ATM	**0.88**	**1.00**	0.67
	Both	**0.88**	**1.00**	0.67

[1] Due to conflict of interest.

References

J. Li, K. Yanagisawa, M. Sugita, T. Fujie, M. Ohue, and Y. Akiyama. Cycpeptmpdb: a comprehensive database of membrane permeability of cyclic peptides. *Journal of Chemical Information and Modeling*, 63(7):2240–2250, 2023.

J. Phan, Z. Li, A. Kasprzak, B. Li, S. Sebti, W. Guida, E. Schönbrunn, and J. Chen. Structure-based design of high affinity peptides inhibiting the interaction of p53 with mdm2 and mdmx. *Journal of Biological Chemistry*, 285 (3):2174–2183, 2010.

J. M. Rogers, T. Passioura, and H. Suga. Nonproteinogenic deep mutational scanning of linear and cyclic peptides. *Proceedings of the National Academy of Sciences*, 115(43):10959–10964, 2018.

S. T. Runyon, Y. Zhang, B. A. Appleton, S. L. Sazinsky, P. Wu, B. Pan, C. Wiesmann, N. J. Skelton, and S. S. Sidhu. Structural and functional analysis of the pdz domains of human htra1 and htra3. *Protein Science*, 16(11):2454–2471, 2007.

M. van Rosmalen, B. M. Janssen, N. M. Hendrikse, A. J. van der Linden, P. A. Pieters, D. Wanders, T. F. de Greef, and M. Merkx. Affinity maturation of a cyclic peptide handle for therapeutic antibodies using deep mutational scanning. *Journal of Biological Chemistry*, 292(4):1477–1489, 2017.
